# Interocular Symmetry of Fixation, Optic Disc, and Corneal Astigmatism in Bilateral High Myopia: The Shanghai High Myopia Study

**DOI:** 10.1167/tvst.8.1.22

**Published:** 2019-02-13

**Authors:** Xiangjia Zhu, Wenwen He, Yu Du, Keke Zhang, Yi Lu

**Affiliations:** 1 Department of Ophthalmology, Eye and Ear, Nose, and Throat Hospital, Funan University, Shanghai, China; 2 Eye Institute, Eye and Ear, Nose, and Throat Hospital of Fudan University, 83 Fenyang Road, Shanghai, People's Republic of China; 3 NHC Key Laboratory of Myopia, Fudan University, 83 Fenyang Road, Shanghai, People's Republic of China; 4 Key Laboratory of Myopia, Chinese Academy of Medical Sciences, 83 Fenyang Road, Shanghai, People's Republic of China; 5 Shanghai Key Laboratory of Visual Impairment and Restoration, Shanghai, People's Republic of China

**Keywords:** high myopia, interocular symmetry, fixation, optic disc, corneal astigmatism

## Abstract

**Purpose:**

We investigate the interocular symmetry of fixation, optic disc, and corneal astigmatism in bilateral high myopia, and evaluate the predictive relationships between them.

**Methods:**

We enrolled 202 cases with bilateral high myopia. Fixation, in terms of the bivariate contour ellipse area (BCEA), was evaluated with the Macular Integrity Assessment microperimetry. Optic disc features, including orientation, tilt, and rotation, were evaluated with ultrawide-field retinal photographs. Corneal topography was performed with Pentacam. Interocular symmetry of fixation, optic disc, and corneal astigmatism was assessed, and the predictive relationships between these parameters were investigated.

**Results:**

Axial length differences between the two eyes were: ≥0 to ≤1 mm, 67.8%; 1 to ≤2 mm, 20.3%; 2 to ≤3 mm, 9.4%; and >3 mm, 2.5%. Axial length, 95% BCEA, and magnitude of corneal astigmatism showed good interocular symmetry, whereas the optic disc tilt, rotation, and axis of corneal astigmatism (mirror axes) showed less symmetry (all *P* < 0.05). No interocular symmetry was observed in the direction of the fixation ellipse. In both eyes, the corneal steep meridian more often was consistent with the optic disc orientation than inconsistent (right eye [OD], *P* < 0.001; left eye [OS], *P* = 0.029).

**Conclusions:**

As different parameters presented different degrees of symmetry, cautions are needed when including both eyes or only one lateral eye in cases of bilateral high myopia for clinical investigations. The optic disc orientation, to some extent, may indicate the steep meridian of the cornea.

**Translational Relevance:**

Our study provided evidences for selection of eye laterality in clinical investigations of highly myopic eyes.

## Introduction

High myopia is a major worldwide concern today, especially in East Asia.^1^–^3^ With elongation of the eyeball, mechanical stretching of the posterior pole may cause degenerative changes in the retina and variations in the position and shape of the optic disc, which consequently lead to functional changes in highly myopic eyes.^2^,^4^,^5^

Several studies have investigated variations in optic disc features, such as tilt, rotation, and parapapillary atrophy, in highly myopic eyes.^2^,^6^–^9^ Most have been focused on the relationships between the morphologic changes in the optic disc and the risk of glaucoma.^7^,^9^ Similar to the optic disc, corneas of highly myopic eyes also show great interindividual variability in their shapes and biomechanical features.^10^,^11^

Previously, we identified an axial length–dependent reduction in fixation stability of highly myopic eyes using the Macular Integrity Assessment (MAIA) microperimetry system (Centervue, Padova, Italy).^12^ The bivariate contour ellipse area (BCEA), which represents the area of the ellipse containing most of the fixation positions registered during the measurement procedure, was used to evaluate fixation stability.^12^,^13^ We also noted that the fixation ellipse angle, defined as the orientation of the longest fixation ellipse diameter, varied even more greatly in these eyes.

Such great variations in these anatomical or functional parameters of highly myopic eyes may inevitably increase the interocular asymmetry in patients with bilateral high myopia. We normally expect eyes and visual systems to behave somewhat symmetrically, and accordingly, we include both eyes of patients for many clinical investigations. However, this may not be correct, especially in highly myopic eyes, which show great structural viability due to abnormal elongation of the eyeballs. Studies have reported a consistent pattern of asymmetry between our eyes. The Sydney Childhood Eye Study found only a low level of interocular correlation in the thickness of the retinal nerve fiber layer (RNFL), with differences of up to 17 μm between the two eyes of normal children.^14^ A study of healthy adults also confirmed that the RNFL is thicker in right eyes.^15^ Thus, it also may be meaningful to investigate the interocular asymmetry of anatomical or functional parameters, such as fixation, optic disc, and corneal astigmatism, in bilateral highly myopic cases.

Additionally, we assume that if the eyeball is an ideal optical system, certain kinds of predictive relationships may exist between the cornea astigmatism median and optic disc orientation or the direction of the macular fixation ellipse, to maintain the coordination of eye function. A previous study showed that an abnormally shaped optic disc can be correlated with abnormal corneal astigmatism and that the steep meridian of corneal astigmatism may reflect the orientation of the longest disc diameter.^10^ Therefore, we also investigated the predictive relationships between fixation, optic disc, and corneal astigmatism.

## Methods

The Shanghai High Myopia Study is a hospital-based prospective cohort study that continuously includes highly myopic patients scheduled for cataract surgery in the Eye and Ear, Nose, and Throat (EENT) Hospital of Fudan University since October 2015. All subjects underwent detailed preoperative examinations and postoperative follow-ups. This investigation is a subproject of the Shanghai High Myopia Study. Individuals with bilateral axial lengths >26 mm were selected for this study; excluded were those whose eyes had severe myopic fundus pathologies, such as choroidal neovascularization, macular hole, or severe macular atrophy, a history of other anterior segment ocular diseases, trauma, or a systemic condition that affected their visual performance. Ultimately, 202 subjects were eligible for this study. The institutional review board of the EENT Hospital of Fudan University approved the study protocol. All procedures were performed in accordance with the tenets of the Declaration of Helsinki. All participants were informed of the study objectives, and each signed an informed consent form to participate in this study. This study was registered at www.clinicaltrials.gov (accession number NCT03062085).

### Preoperative and Postoperative Examinations

Preoperative examinations included assessment of visual acuity, tonometry, measurement of axial length (IOLMaster 500, Version 7.7; Carl Zeiss AG, Jena, Germany), funduscopy, B scans, and macular scans with optical coherence tomography (OCT; Zeiss Cirrus HD-OCT 5000; Carl Zeiss AG). Corneal topography was performed with the Pentacam system (Pentacam HR, Oculus Optikgeräte GmbH, Wetzlar, Germany), and the steep meridian of the anterior corneal astigmatism was recorded. Follow-up included assessment of visual acuity, manifest refraction, tonometry, funduscopy, retinal photography, MAIA microperimetry, and an OCT macular scan.

### Fixation Assessment

Fixation was assessed using the MAIA microperimetry system. The patients were asked to stare at the fixation stimulus, which consisted of a red circle with a diameter of 1°. The eye trackers within the MAIA system detected fixation loss as a misalignment of the directions of the central fixation stimulus and gaze, and recorded the points of fixation. The device then automatically calculated two parameters of fixation: the BCEA, which refers to the area (in degrees squared, deg^2^) of the ellipse containing most of the fixation positions registered during the measurement procedure, and the fixation ellipse angle, the orientation of the longest ellipse diameter. BCEA normally is calculated by considering 63% or 95% of the fixation points. Because these two parameters are similar, only 95% BCEA was used for further analysis in this study.

### Measurements of Optic Disc Tilt and Rotation

**Figure 1:**
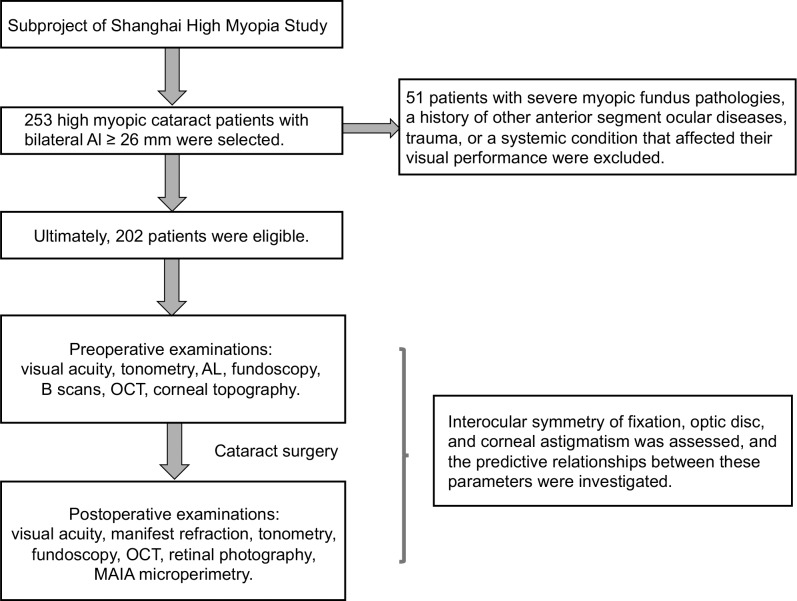
Flow chart of the study. AL, axial length.

Ultrawide-field retinal images were obtained with a nonmydriatic ultrawide-field scanning laser ophthalmoscope (Optomap 200Tx; Optos Plc., Dunfermline, Scotland) with standard settings. The measurements of optic disc tilt and rotation were obtained as described previously.^4^,^16^ Optic disc tilt was defined as the ratio between the longest and shortest diameters of the optic disc, and those with ratios >1.30 were considered tilted. Rotation was measured as the deviation of the long axis of the optic disc from a reference line at 90° from a horizontal line connecting the fovea and center of the optic disc. The angle between the long axis of the optic disc and the reference line was defined as the degree of rotation, and rotation >15° was deemed significant. Superior rotation is shown as a negative value and inferior rotation as a positive value. The orientation of the longest disc diameter also was recorded for direct comparison with the fixation ellipse angle and steep meridian of corneal astigmatism. Figure 1 displays the flow chart of the study.

### Statistical Analysis

The agreement between two observers on the optic disc orientation, tilt ratio, and degree of rotation was evaluated with the Bland–Altman method, which plots their mean results against their differences (Supplementary Fig. S1). The limit of agreement was calculated as the mean difference in two measurements ± 1.96 standard deviations (SD) of the difference, according to previous studies.^4^,^7^ Data were analyzed with SPSS version 11.0 (SPSS, Inc., Chicago, IL) and all data are shown as means ± SD. A Student's *t*-test was used to compare continuous variables and the χ^2^ test was used to compare categorical variables of two eyes. Intraclass correlation coefficients (ICC) and Pearson's coefficients were computed to measure the interocular agreement or correlation. The relationships between the cumulative distribution functions were assessed with the Kolmogorov–Smirnov test. *P* < 0.05 was considered statistically significant.

## Results

**Figure 2:**
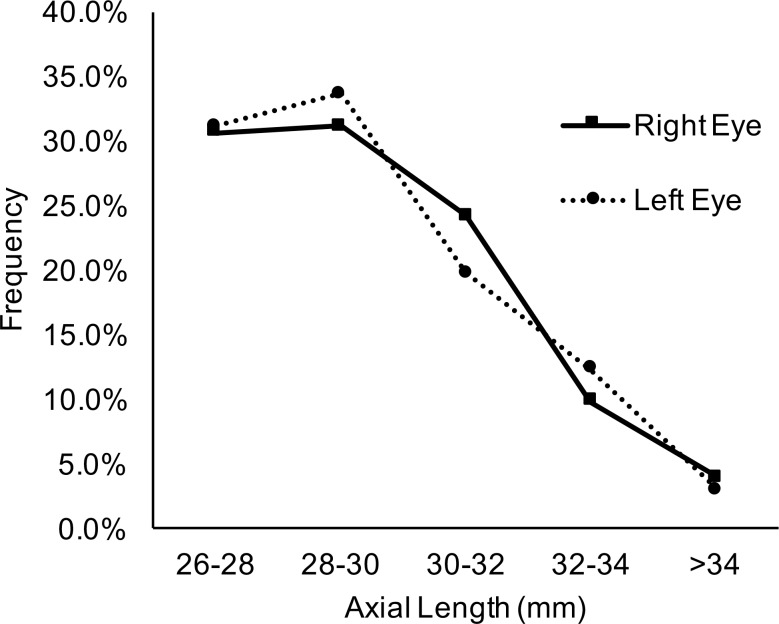
Distribution of the ALs in both eyes indicating the distribution of myopia severity.

The characteristics of the subjects are shown in Table 1. Average age was 61.41 ± 8.56 years; 81 subjects were male and 121 were female. No statistically significant differences were found between the left and right eyes in terms of uncorrected (UCVA) and best corrected (BCVA) visual acuity, axial length, intraocular pressure, 95% BCEA, fixation ellipse angle, optic disc rotation, corneal astigmatism, or steep corneal meridian (Student's *t-*test, all *P* > 0.05), except that the left eyes had significantly higher optic tilt ratios than the right eyes (1.31 ± 0.22 vs. 1.36 ± 0.24, Student's *t*-test, *P* = 0.002). Figure 2 shows the distribution of axial lengths in both eyes indicating the distribution of myopia severity.

**Table 1:** Demographic Data for the Participants

Parameter	Value	*P* Value
Age, year	61.41 ± 8.56	-
Sex (male/female)	81/121	-
Right eye/Left eye:		
UCVA, logMAR	1.19 ± 0.67/1.16 ± 0.64	0.644
BCVA, logMAR	0.86 ± 0.64/0.82 ± 0.57	0.447
AL, mm	29.43 ± 2.40/29.31 ± 2.39	0.587
IOP, mm Hg	15.48 ± 3.20/15.43 ± 3.33	0.965
95%BCEA, deg^2^	17.60 ± 24.71/15.10 ± 17.37	0.242
Fixation ellipse angle, °	84.56 ± 52.57/89.54 ± 55.45	0.314
Optic disc tilt		
Tilted disc, no (%)	102 (50.5) /115 (56.9)	0.195
Tilt ratio	1.31 ± 0.22/1.36 ± 0.24	0.010^a^
Optic disc rotation		
Superior, no (%)	63 (31.2)/64 (31.7)	0.915
Inferior, no (%)	139 (68.8)/138 (68.3)	0.915
Significant, no (%)	87 (43.1)/97 (48.0)	0.318
Rotation degree, °	6.34 ± 29.88/10.19 ± 31.55	0.209
Corneal astigmatism, D	1.04 ± 0.85/1.13 ± 0.71	0.272
Steep corneal meridian, °	92.91 ± 50.74/96.08 ± 47.87	0.504

IOP, intraocular pressure.

a Difference in the tilt ratio between the two eyes was significant (Student's *t-*test).

**Figure 3:**
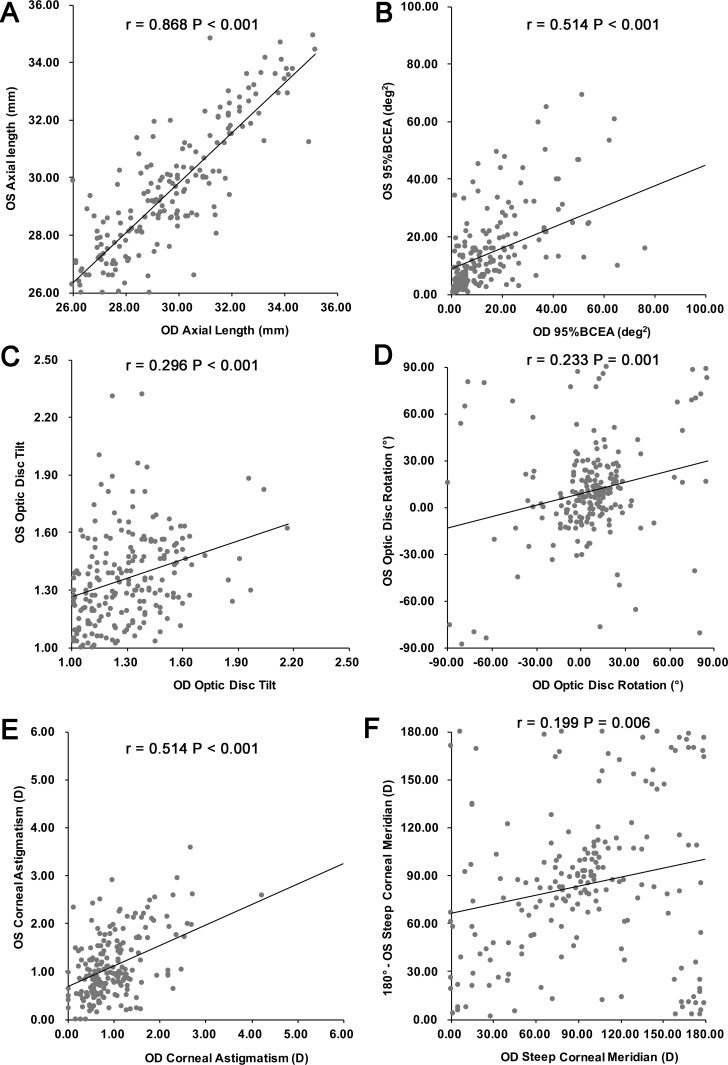
Correlation of AL (A), 95% BCEA (B), optic disc tilt ratio (C), optic disc rotation (D), magnitude of corneal astigmatism (E), and steep corneal meridian (F) between the right and left eyes. All parameters correlated positively between eyes, except the steep corneal meridian, which had a positive mirror correlation (Pearson's correlation, all P < 0.05).

We firstly investigated interocular symmetry. The distributions of the differences in axial length between the two eyes were: ≥0 to ≤1 mm, 67.8% (137/202); 1 to ≤2 mm, 20.3% (41/202); 2 to ≤3 mm, 9.4% (19/202); and >3 mm, 2.5% (5/202). Table 2 presents the interocular differences in different parameters (ICC analysis). High interocular symmetry was observed for axial length, 95% BCEA, and magnitude of corneal astigmatism (ICC analysis, all *P* < 0.05). The optic disc tilt, rotation and steep corneal meridian (axis of the right eye and mirror axis of the left eye) showed low to moderate correlations (ICC analysis, all *P* < 0.05), indicating interocular asymmetry increased. The findings were similar with Pearson's correlation analysis (Fig. 3). However, no interocular symmetry was detected for the direction of the fixation ellipse with either statistical method (Pearson's analysis, *r* = 0.017, *P* = 0.808; ICC = 0.034, *P* = 0.40).

**Table 2:** ICC Analysis of the Ocular Characteristics of the Right and Left Eyes

Parameter	ICC (95% CI)	*P* Value
AL	0.929 (0.906–0.947)	<0.001
95% BCEA	0.650 (0.538–0.734)	<0.001
Fixation ellipse angle	0.034 (−0.275–0.268)	0.404
Optic disc tilt	0.445 (0.271–0.578)	<0.001
Optic disc rotation	0.377 (0.179–0.527)	<0.001
Corneal astigmatism	0.670 (0.562–0.751)	<0.001
Steep corneal meridian^a^	0.328 (0.109–0.493)	0.003

ICC, intraclass correlation coefficients; CI, confidence interval.

a Steep corneal meridian was compared in the axis of the right eye and the mirror axis of the left eye.

**Figure 4:**
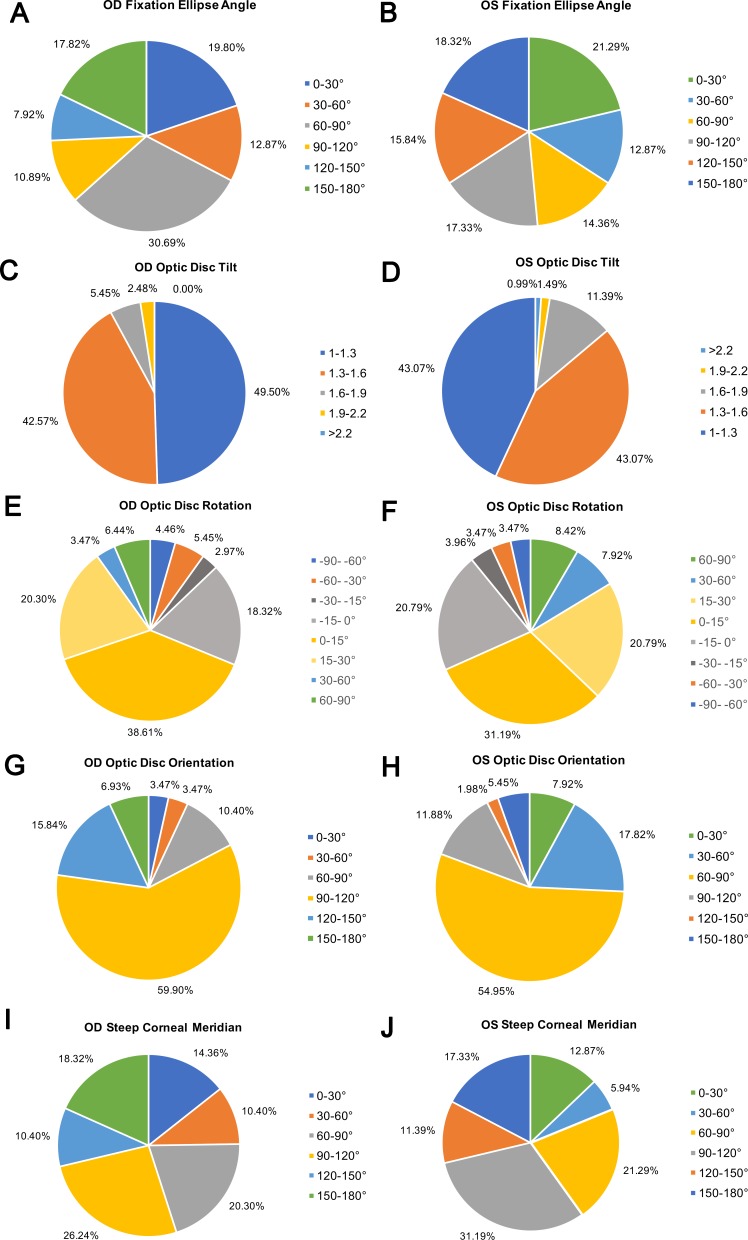
Distributions of the fixation ellipse angle (A, B), optic disc tilt (C, D), and rotation (E, F), orientation of the longest disc diameter (G, H), and steep corneal meridian (I, J) in the right and left eyes. The paired subfigures are colored as mirror images.

Figure 4 presents the distributions of the fixation ellipse angles, optic disc tilt and rotation, orientations of the longest disc diameters, and steep corneal meridians in the right and left eyes. The paired subfigures are colored as mirror images. The fixation angle was distributed almost evenly in all directions in both eyes; 50.50% of the right and 56.93% of left eyes had a tilted disc. Significant rotation of the disc occurred in 43.07% (87/202) of right and 48.02% (97/202) of left eyes. Inferior rotation was present in 68.8% and 68.3%, respectively. With regard to the orientation of the longest disc diameter, 59.9% of the right eyes were located between 90° and 120° and 15.84% between 120° and 150°, while 54.95% of the left eyes were located between 60° and 90° and 17.82% between 30° and 60°, suggesting both eyes presented inferior rotation in a symmetrical way.

**Figure 5:**
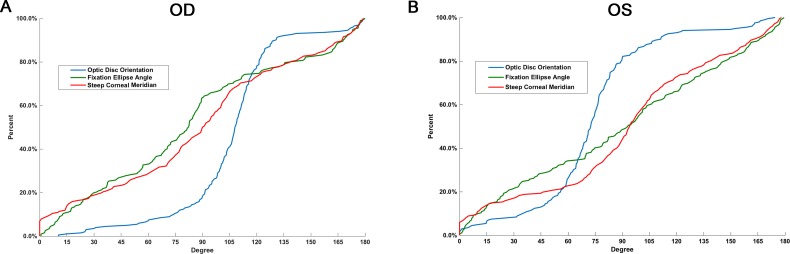
Cumulative distribution function curves for optic disc orientation, fixation ellipse angle, and steep corneal meridian in the right (A) and left (B) eyes. Distribution pattern of steep corneal meridian seemed more similar to that of the fixation ellipse angle than to the distribution of the optic disc orientation. However, statistically significant differences were found in their distributions (Kolmogorov–Smirnov test, cornea vs. fixation: right eye, P = 0.003; left eye, P = 0.021; cornea vs. disc, both eyes, P < 0.001).

From the cumulative distribution function curves shown in Figure 5, the distribution pattern of the steep corneal meridian seemed more similar to that of the fixation ellipse angle than to that of the optic disc orientation. However, statistically significant differences were found in their distributions (Kolmogorov–Smirnov test: cornea vs. fixation, right eye, *P* = 0.003; left eye, *P* = 0.021; cornea vs disc, both eyes, *P* < 0.001).

We then evaluated the predictive relationships between the direction of the fixation ellipse, orientation of the longest disc diameter, and steep corneal meridian. We defined 0° to 45° and 135° to 180° as the horizontal zone and 45 to 135° as the vertical zone. If the steep corneal meridian and optic disc orientation or the fixation ellipse angle were located in the same zone, their directions were considered consistent. The steep corneal meridian more often was consistent with the optic disc orientation than inconsistent in the right and left eyes, (*χ*^2^ test, right eye, *P* < 0.001; left eye, *P* = 0.029). However, no such consistency was detected between the steep corneal meridian and fixation ellipse angle in either eyes (*χ*^2^ test, both eyes, *P* = 0.111). In addition, the proportion of the angles between the steep corneal meridian and optic disc orientation that were <10° was 14.4% (29/202) in the right eye and 11.9% (24/202) in the left eye, and the proportion of the angles between the steep corneal meridian and fixation ellipse angle that were <10° was 13.9% (28/202) and 9.4% (19/202), respectively. If the angle range was set at <45°, the percentage was 55.9% (113/202) and 50.5% (102/202), respectively, for the former, and 49.5% (100/202) and 46.5% (94/202), respectively, for the latter.

## Discussion

Symmetry is defined as two or more matching regions that are identical or nearly identical by either mirror or rotational reflection, and it is observed in much of the biological world. As humans, we are familiar with our gross anatomical symmetry and the important asymmetries within us. Our eyes also are thought to be symmetrical organs, and studies reporting asymmetry between the two eyes have emerged after many precise measures of ocular structures have become possible.^15^ High myopia is known to cause large variations in the structure of the eyeball, especially the posterior pole.^4^,^5^,^17^ A number of studies have investigated these variations, focusing on the optic disc tilt and rotation,^4^,^17^ corneal astigmatism,^10^ or structure of posterior staphyloma.^5^ However, very few studies exist of the interocular symmetry of the eye structures in patients with bilateral high myopia, or of the predictive relationships between the cornea and posterior structures within each eye. In this study, different ocular features displayed different degrees of symmetry. Higher interocular symmetry was observed for axial length, 95% BCEA, and magnitude of corneal astigmatism, and lower levels of symmetry were found in the optic disc tilt, rotation, and steep corneal meridian. The optic disc orientation was more correlated with the steep corneal meridian than was the fixation ellipse angle.

Although the axial length, 95% BCEA, and magnitude of corneal astigmatism showed relatively good interocular symmetry, extensive asymmetry is present in bilateral high myopia. With advances in biometry, interocular differences have been identified in the thickness of RNFLs,^14^,^15^ morphometry of the retinal vasculature,^18^ and the optic nerve, and so forth. The RNFL is systematically thicker in right eyes, and this has been verified in children aged 6 years^14^ and in adults.^15^ Hwang et al.^19^ also found significant interocular differences in all quadrants of the circumpapillary RNFL in adults. A Turkish study demonstrated that the optic nerve sheath diameter in the left eyes of a healthy population was significantly greater than that in the right eyes.^20^ Ocular dominance also had an influence on ocular torsion and in a way that decreases the torsion in the dominant eye^21^ and the right eye is the dominant eye in most people.^22^ Likewise, in our study, a significantly higher optic tilt ratio was detected in left eyes. Further investigation with the ICC method and Pearson's correlation also revealed interocular asymmetry for optic disc tilt and rotation. The correlation coefficients were <0.5, which were clearly lower than the coefficient of 0.77 reported by the Blue Mountains Eye Study when retinal vessel symmetry was examined in a normal elderly population.^23^ These findings suggest that greater interocular asymmetry exists in the posterior segment of bilateral high myopia than in normal eyes.

Previous studies have defined two types of symmetry for corneal astigmatism: direct (equal axes) and mirror (mirror axes) symmetry. McKendrick et al.^24^ reported that neither type of symmetry was predominant, whereas Guggenheim et al.^25^ reported that the astigmatism axes of fellow eyes tended to display mirror symmetry. According to our study, in patients with bilateral high myopia, magnitude of corneal astigmatism showed a high degree of symmetry, whereas the steep corneal meridian was relatively asymmetric.

Fixation is a recently proposed concept in the study of ocular function. In healthy eyes, small fixational eye movements in the right and left eyes are not absolutely symmetrical, and this can be quantified using the BCEA measured with MAIA microperimetry.^26^ Fixation is now accepted as an indicator of visual function.^27^ However, few related studies have been conducted in the highly myopic population. Our study demonstrated that bilateral high myopia presented good symmetry in BCEA, but very low symmetry in the angle of fixation ellipse, suggesting that consistent bilateral fixation stability is achieved probably to guarantee the proper and coordinated visual functions of the two eyes, while the inconsistency of direction may be due to the anatomic or pathologic variations in the posterior poles of bilateral high myopia.

The structural and functional symmetry in highly myopic eyes has important clinical significance. High myopia is an oculopathy that causes a series of anatomic and pathologic changes, which may consequently induce greater asymmetry between the right and left eyes. This possibility challenges the reliability of studies that enroll only one lateral of eyes or those that mixed the data of two eyes. Bias also will be introduced that impairs the reference values for ocular parameters taken from normative databases. Bilateral asymmetry in ocular metrics also might affect the occurrence of certain ocular diseases. There is asymmetry in the timing of progression of age-related macular degeneration, as reported by the researchers in the Beaver Dam Eye Study.^28^ Similarly, glaucoma, although typically a bilateral disease, shows pervasive asymmetry in its early stages. A difference in the cup–disc ratio of > 0.2 between eyes is a feature that suggests glaucoma.^29^ Interocular asymmetry in the optic nerve and macular metrics is also more useful in diagnosing glaucoma than data from only one lateral eye.^30^ Taking these findings together, it is likely that in cases of bilateral high myopia, with excessive elongation of the eyeball adding to ocular structural variations, symmetry and asymmetry studies are important to precisely predict and evaluate the complications that aggravate patients' visual function.

**Figure 6:**
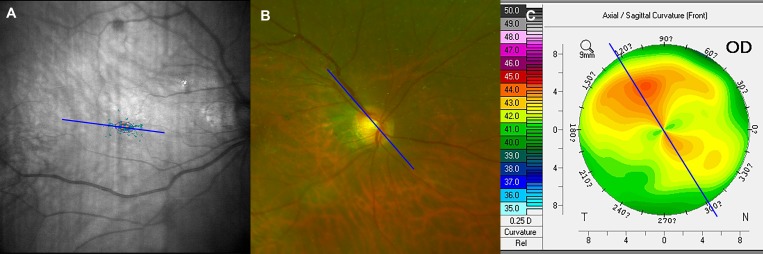
Fixation ellipse angle (A), optic disc orientation (B) and steep corneal meridian (C) of a representative highly myopic case. The longest disc diameter provided a more reliable indication of the steep corneal meridian than the fixation ellipse angle.

The predictive relationships between the anterior and posterior poles of the eyeball also are important, because they affect the coordination of the optical system, as in architectonics, in which a correspondence between the roof and ground floor structures is required. A previous study proposed that the steep corneal meridian is associated with the orientation of the longest disc diameter;^10^ however, their study population was not highly myopic. Besides, the fixation ellipse angle, being a functional parameter, may be a better corresponding index to corneal steep meridian than the orientation of optic disc. Therefore, we explored the relationships between the corneal astigmatism, optic disc, and macula. We found that, notwithstanding the seemingly similar distributions of the steep corneal meridian and fixation ellipse angle, the longest disc diameter provided a more reliable indication of the steep corneal meridian (Fig. 6), indicating that we may, to some extent, judge steep corneal meridian from the orientation of optic disc with a funduscope. As far as we know, these associations have not been investigated previously.

To conclude, in patients with bilateral high myopia, different parameters presented different degrees of symmetry. Therefore, cautions are needed in interpreting data from clinical investigations, which include both eyes or only one lateral eye in cases of bilateral high myopia. The optic disc orientation may, to some extent, indicate the steep meridian of the cornea.

## Supplementary Material

Supplement 1
