# Supplementary material for: Interocular Symmetry of Fixation, Optic Disc, and Corneal Astigmatism in Bilateral High Myopia: The Shanghai High Myopia Study
Source: Transl Vis Sci Technol. 2019 Feb 13;8(1):22. doi: 10.1167/tvst.8.1.22 (PMC6375117; doi:10.1167/tvst.8.1.22)
Supplement: Supplement 1 [file tvst-08-01-11_s01.pdf]

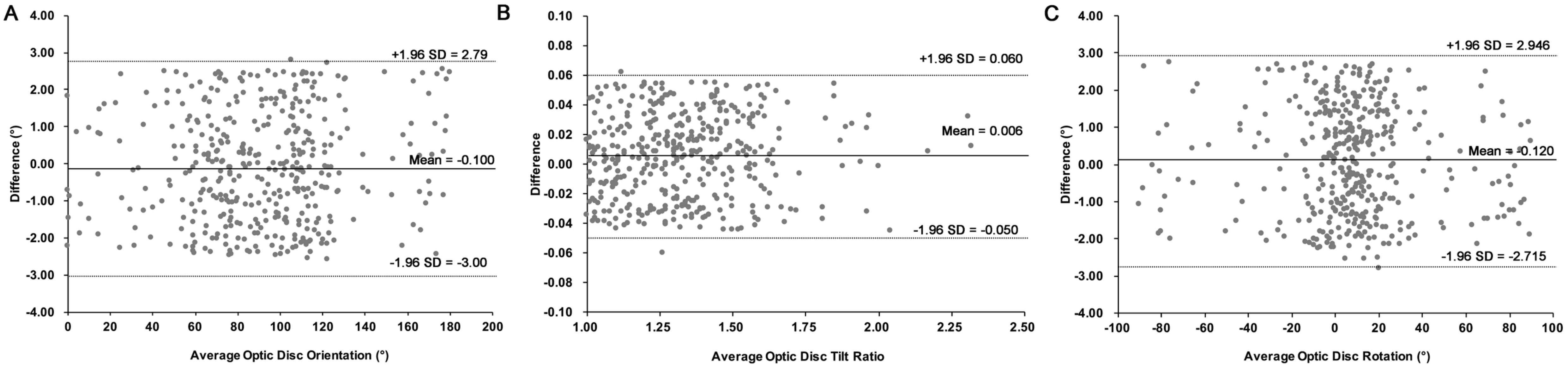


Supplementary Figure S1. Bland–Altman plots showing the agreement in optic disc orientation (A), optic disc tilt ratio (B), and the degree of optic disc rotation (C) measured by two researchers. Reference lines correspond to the mean difference and 95% confidence intervals of the differences. The agreement in the parameters was good, and there was no systemic bias in the measurements. SD, standard deviation.
